# Large-scale ruthenium- and enzyme-catalyzed dynamic kinetic resolution of (*rac*)-1-phenylethanol

**DOI:** 10.1186/1860-5397-3-50

**Published:** 2007-12-20

**Authors:** Krisztián Bogár, Belén Martín-Matute, Jan-E Bäckvall

**Affiliations:** 1 Department of Organic Chemistry, Arrhenius Laboratory, Stockholm University, SE-106 91 Stockholm, Sweden

## Abstract

The scale-up of the ruthenium- and enzyme-catalyzed dynamic kinetic resolution (DKR) of (*rac*)-1-phenylethanol (**2**) is addressed. The immobilized lipase *Candida antarctica* lipase B (CALB) was employed for the resolution, which shows high enantioselectivity in the transesterification. The ruthenium catalyst used, (*η* ^5^-C_5_Ph_5_)RuCl(CO)_2_ **1**, was shown to possess very high reactivity in the "*in situ*" redox racemization of 1-phenylethanol (**2**) in the presence of the immobilized enzyme, and could be used in 0.05 mol% with high efficiency. Commercially available isopropenyl acetate was employed as acylating agent in the lipase-catalyzed transesterifications, which makes the purification of the product very easy. In a successful large-scale DKR of **2,** with 0.05 mol% of **1,** (*R*)-1-phenylethanol acetate (**3**) was obtained in 159 g (97% yield) in excellent enantiomeric excess (99.8% ee).

## Background

Chiral alcohols are important synthetic intermediates and are structural elements in biologically active compounds and natural products. [1–4] A few methods have been developed for the enantioselective synthesis of chiral alcohols including catalytic asymmetric hydrogenation [5–14] and enantioselective hydride addition [15–16] of prochiral ketones, asymmetric dialkylzinc addition to aldehydes, [17–18] and asymmetric aldol reactions. [19–23] Biocatalysts have also been successfully employed in the enantioselective reduction of ketones.[24] The enzymatic resolution of racemic alcohols is a convenient alternative for preparing enantiomerically pure alcohols,[1,25–28] and recently this approach has been extended to dynamic kinetic resolution (DKR) by combining the enzymatic resolution with a metal-catalyzed racemization. [29–37] Today, many stable lipases are commercially available and they are frequently used in synthetic organic chemistry.[27]

**Figure 1:**
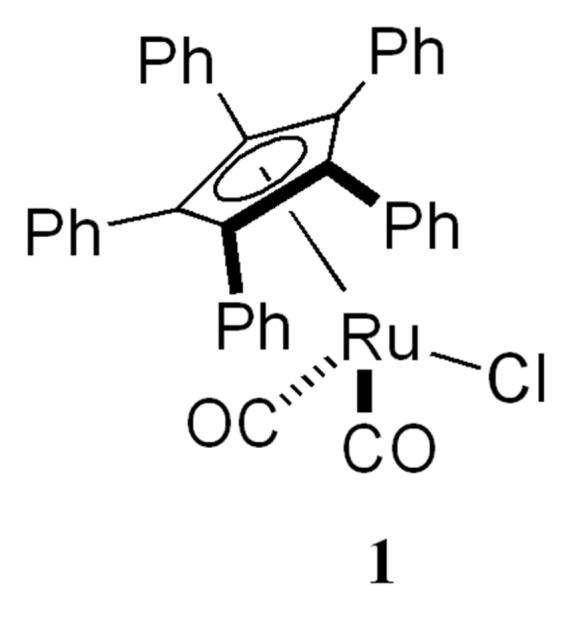
Racemization catalyst **1**.

In the course of the chemo-enzymatic synthesis of optically pure alcohols and esters, *Candida antarctica* lipase B (CALB) was found to be one of the most active and selective biocatalysts compared to other enzymes. Lipases do not need the presence of a cofactor and they can be employed in pure organic solvents.[38–39] CALB is immobilized on polyacrylate and this increases its thermostability and makes it easy to recover from the reaction mixture. Recently, we have developed highly efficient DKR protocols for secondary alcohols in which the traditional enzymatic resolution is combined with an "*in situ*" racemization of the substrate using a ruthenium(II) racemization catalyst (**1**) at ambient temperature (Figure 1).[32,34]

Many 1-arylethanols are used as intermediates for the synthesis of pharmaceuticals and agrochemicals,[40–41] and they are therefore needed in enantiomerically pure form. Herein, we address some of the scale-up issues of the ruthenium- and enzyme-catalyzed DKR of (*rac*)-1-phenylethanol under mild conditions.

## Results and Discussions

**Scheme 1:**
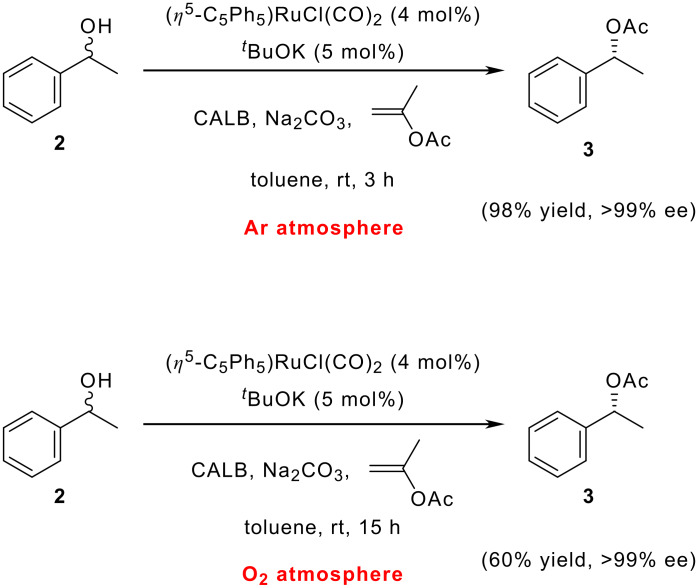
DKR of 1-phenylethanol under an Ar atmosphere (top) and DKR of 1-phenylethanol under an O_2_ atmosphere (bottom).

The DKR of 1-phenylethanol has been tested under different reaction conditions with the aim of decreasing the catalyst loading. When the reaction is performed on a 1 mmol scale, 4 mol% of ruthenium complex **1** is used for an efficient reaction.[32,34] We believe that the need of this high catalyst loading is due to a fast decomposition of the ruthenium active intermediates in the presence of small amounts of molecular oxygen. To test this hypothesis, the DKR of 1-phenylethanol under argon was compared to the DKR of 1-phenylethanol (**2**) under an oxygen atmosphere (Scheme 1).

While 98% of enantiomerically pure (>99% ee) (*R*)-1-phenylethanol acetate (**3**) was obtained in only 3 hours under an argon atmosphere, only 60% of **3** was produced after 16 hours under an oxygen atmosphere. We envisioned that running the reaction on a larger scale would allow a decrease in the catalyst loading, since decomposition of the catalyst due to the presence of traces of molecular oxygen may be avoided. The reaction in Scheme 1 (under argon) was therefore run on a larger scale and the results are summarized in Table 1. On a 10 mmol-scale, 1 mol% of Ru-catalyst **1** can be used to achieve 90% yield of acetate **3** (99% ee) after 18 h at room temperature (entry 1). A decrease of the catalyst loading to 0.5 mol% afforded 87% of enantiopure acetate, although a longer reaction time was required (42 h, entry 2). A significant improvement was obtained when the reaction was carried out at 40°C (entry 3). At this temperature, 0.5 mol% of catalyst **1** is sufficient to obtain a quantitative yield (99.5%) of the enantiomerically pure acetate **3** (>99% ee) within 18 h. The very high efficiency of the latter reaction suggests that the racemization catalyst loading can be further decreased, and that an increased reaction temperature may facilitate this decrease.

**Table 1:** Dynamic kinetic resolution of 1-phenylethanol (**2**) under different reaction conditions.^a^

Entry	**2** (mmol)	[Ru] (mol%)	^t^BuOK (mol%)	CALB (mg/mmol)	Na_2_CO_3_ (mg/mmol)	T (°C)	Ratio **2**/solv (w/w)	time	Yield of **3** (%)	ee of **3** (%)^b^

1	10	1	1.5	2	50	rt	0.14	18 h	90^c^	>99
2	10	0.5	0.75	2	25	rt	0.28	42 h	87^c^	>99
3	10	0.5	0.75	2	25	40	0.28	18 h	99.5^c^	>99
4	1000	0.05	0.075	0.5	21.2	70	0.94	20 h	97^d^	>99
5	10000	0.01	0.015	0.1	3	70	20.1	21 d	87^d^	97

a. Toluene was used as solvent. b: ee measured by GLC; c: Yield measured by GLC; d: Isolated yield by distillation.

Indeed, a DKR reaction at 70°C on a 1 mol-scale employing only 0.05 mol% Ru-catalyst in a more concentrated reaction mixture afforded enantiopure acetate **3** in 97% yield after 20 h. Careful analysis of the enantiomeric purity of acetate **3** revealed that it was >99.8% ee. This result is remarkable since only a very low catalyst loading is employed and in addition, only 150 mL of toluene as the solvent is used to produce 159 g of enantiomerically pure **3**. Finally, to prove the high efficiency of the racemization catalyst **1**, DKR was carried out on a 10 mol-scale employing lower amount of metal- and biocatalyst in a more concentrated reaction mixture. After 21 days, enantiomerically enriched acetate **3** (97% ee) was obtained in 87% yield (corresponds to 1.43 kg). The remaining alcohol had an ee of 19%, demonstrating the high racemization ability activity of ruthenium complex **1**.

## Conclusion

In this article, some of the scale-up issues in the ruthenium- and enzyme-catalyzed DKR of (*rac*)-1-phenylethanol were investigated. Under optimized reaction conditions, DKR of 1-phenylethanol (**2**) was performed delivering 159 g (97% yield) of enantiomerically pure (*R*)-1-phenylethanol acetate (**3**) in a short reaction time (20 h) using 0.05 mol% of Ru-catalyst **1**, and small amounts of enzyme. The employed heterogeneous biocatalyst, *Candida antarctica* lipase B, catalyzes the transesterification with excellent selectivity in the presence of the homogeneous ruthenium racemization catalyst **1**. Isopropenyl acetate seems to be an appropriate acyl donor, which makes the purification of the product acetate very easy via simple distillation.

## Experimental

See Supporting Information File 1 for experimental data.

## Supporting Information

File 1 Large-scale ruthenium- and enzyme-catalyzed dynamic kinetic resolution of (*rac*)-1-phenylethanol: Supporting Information. Experimental procedure of DKR of **2** on 1 mol scale and GLC chromatogram of this experiment.
