# Supplementary material for: Large-scale ruthenium- and enzyme-catalyzed dynamic kinetic resolution of (rac)-1-phenylethanol
Source: Beilstein J Org Chem. 2007 Dec 20;3:50. doi: 10.1186/1860-5397-3-50 (PMC2200671; doi:10.1186/1860-5397-3-50)
Supplement: File 1 — Large-scale ruthenium- and enzyme-catalyzed dynamic kinetic resolution of (rac)-1-phenylethanol: Supporting Information. Experimental procedure of DKR of 2 on 1 mol scale and GLC chromatogram of this experiment. [file Beilstein_J_Org_Chem-03-50-s001.doc]

**Supporting Information**

**Large scale ruthenium- and enzyme-catalyzed dynamic kinetic resolution of (*rac*)-1-phenylethanol**

**Krisztián Bogár, Belén Martín-Matute and Jan-E. Bäckvall***

Department of Organic Chemistry, Arrhenius Laboratory, Stockholm University, SE-106 91 Stockholm, Sweden

[jeb@organ.su.se](mailto:jeb@organ.su.se)

[belen@organ.su.se](mailto:belen@organ.su.se)

[Krisztian.Bogar@astrazeneca.com](mailto:Krisztian.Bogar@astrazeneca.com)

## Experimental

Ruthenium catalyst **1** was prepared as described in ref 10c. Immobilized CALB (Novozym-435) was purchased from Sigma Aldrich.

**Dynamic kinetic resolution of 1-phenylethanol (2) on a 1 mol-scale.** Ru-catalyst **1** (320 mg, 0.5 mmol), immobilized CALB (500 mg), and Na_2_CO_3_ (21.2 g, 0.2 mol) were placed in a 500 mL two-necked round-bottomed flask equipped with a magnetic stirring bar. The flask was evacuated and filled with argon. Dry toluene (150 mL) was added under an argon atmosphere. The reaction mixture was stirred at room temperature until the ruthenium complex was dissolved, and then a solution of *^t^*BuOK in THF (0.5 M) (1.5 mL, 0.75 mmol) was added. The reaction mixture was stirred for 6 min. Then 1-phenylthanol (**2**) (122 g, 1 mol) was added via syringe and the mixture was stirred for another 4 min. Finally, isopropenyl acetate (120 g, 1.2 mol) was added via syringe, and the mixture was stirred at 70 ºC. After 20 h an aliquot (50 μL) was taken and quenched with 1 M HCl. The sample was passed through silica pad and analyzed by GLC equipped with a chiral capillary column (CP-Chirasil-DEX-CB, 25 m*0.32 mm*0.25 μm. Conditions: Temperature: 110 ºC during 12 min; then up to 200 ºC at 80 ºC/min; hold: 6 min. Total time: 19.13 min). The analysis showed that almost no starting material was left. The flask was cooled down to ambient temperature, and the mixture was filtered and concentrated under reduced pressure. The crude mixture was subjected to distillation under reduced pressure (*ca*. 1 Torr) yielding 159 g (97 % yield) of (*R*)-1-phenylethanol acetate **3** (>99.8% ee) as a colorless liquid. Careful analysis by GC showed that the product contained ~1 % of alcohol **2** and <0.5% acetophenone. The latter compound was also present in the commercial racemic **2** that was used.


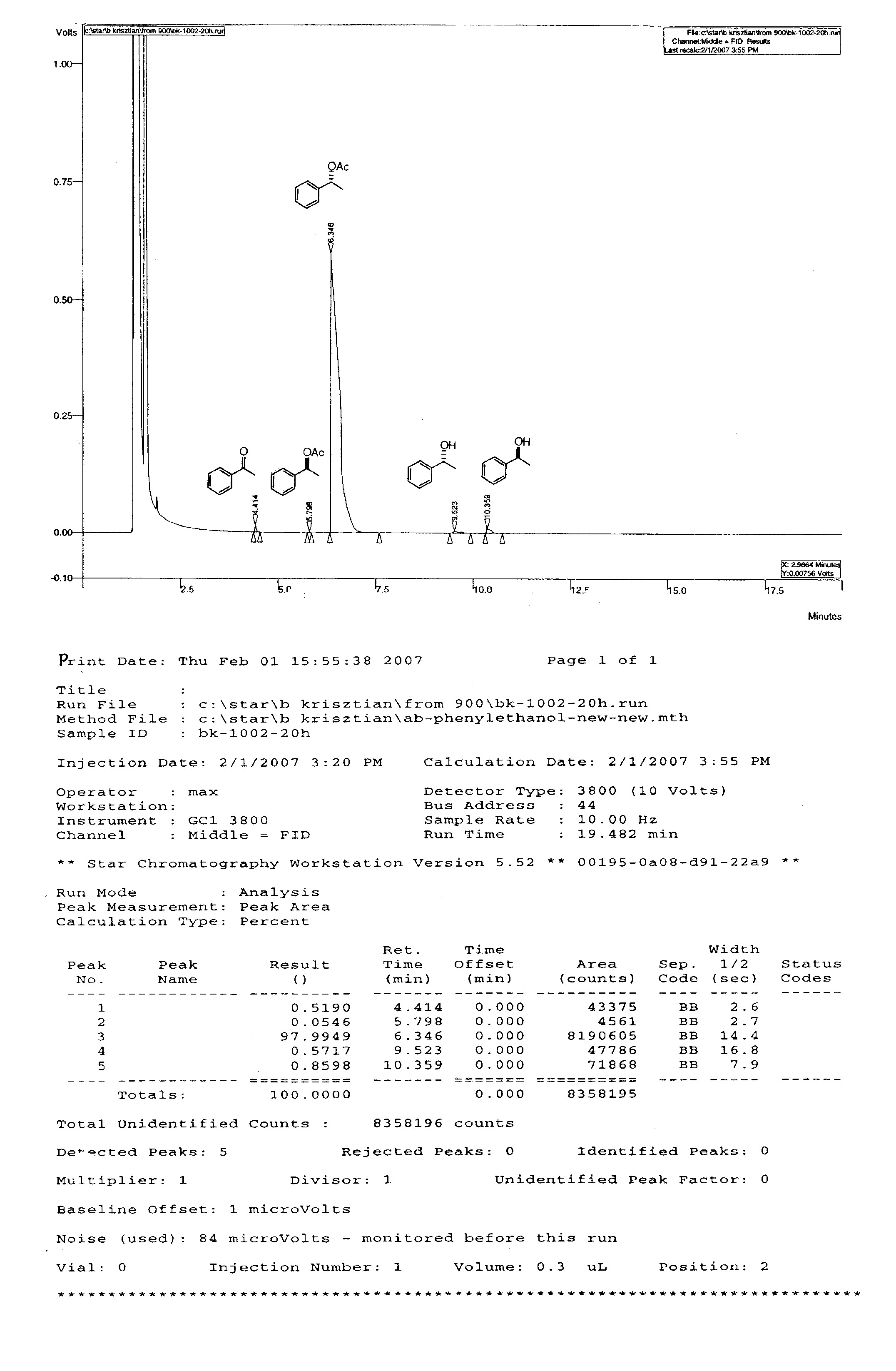


Conditions: CP-Chirasil-DEX-CB, 25 m*0.32 mm*0.25 μm. Conditions: Temperature: 110 ºC during 12 min; then up to 200 ºC at 80 ºC/min; hold: 6 min. Total time: 19.13 min
